# Patterns and determinants of care seeking for obstetric complications in rural northwest Bangladesh: analysis from a prospective cohort study

**DOI:** 10.1186/s12913-015-0832-1

**Published:** 2015-04-18

**Authors:** Shegufta S Sikder, Alain B Labrique, Ian M Craig, Mohammad Abdul Wakil, Abu Ahmed Shamim, Hasmot Ali, Sucheta Mehra, Lee Wu, Saijuddin Shaikh, Keith P West, Parul Christian

**Affiliations:** 1 Department of International Health, Johns Hopkins Bloomberg School of Public Health, Baltimore, MD USA; 2 The JiVitA Maternal and Child Health Research Project, Gaibandha, Bangladesh

**Keywords:** Care seeking, Maternal health, Rural Bangladesh, Cohort study, Global health

## Abstract

**Background:**

In communities with low rates of institutional delivery, little data exist on care-seeking behavior for potentially life-threatening obstetric complications. In this analysis, we sought to describe care-seeking patterns for self-reported complications and near misses in rural Bangladesh and to identify factors associated with care seeking for these conditions.

**Methods:**

Utilizing data from a community-randomized controlled trial enrolling 42,214 pregnant women between 2007 and 2011, we used multivariable multinomial logistic regression to explore the association of demographic and socioeconomic factors, perceived need, and service availability with care seeking for obstetric complications or near misses. We also used multivariable multinomial logistic regression to analyze the factors associated with care seeking by type of obstetric complication (eclampsia, sepsis, hemorrhage, and obstructed labor).

**Results:**

Out of 9,576 women with data on care seeking for obstetric complications, 77% sought any care, with 29% (n = 2,150) visiting at least one formal provider and 70% (n = 5,149) visiting informal providers only. The proportion of women seeking at least one formal provider was highest among women reporting eclampsia (57%), followed by hemorrhage (28%), obstructed labor (22%), and sepsis (17%) (p < 0.001). In multivariable analyses, socioeconomic factors such as living in a household from the highest wealth quartile (Relative Risk Ratio of 1.49; 95% CI of [1.33-1.73]), women’s literacy (RRR of 1.21; 95% CI of [1.05-1.42]), and women’s employment (RRR of 1.10; 95% CI of [1.01-1.18]) were significantly associated with care seeking from formal providers. Service factors including living less than 10 kilometers from a health facility (RRR of 1.16; 95% CI of [1.05-1.28]) and facility availability of comprehensive obstetric services (RRR of 1.25; 95% CI of 1.04-1.36) were also significantly associated with seeking care from formal providers.

**Conclusions:**

While the majority of women reporting obstetric complications sought care, less than a third visited health facilities. Improvements in socioeconomic factors such as maternal literacy, coupled with improved geographic access and service availability, may increase care seeking from formal facilities. Enhancing community awareness on symptoms of hemorrhage, sepsis, and obstructed labor and their consequences may promote care seeking for obstetric complications in rural Bangladesh.

**Trial registration:**

Trial Registration Number: NCT00860470.

## Background

For complications that occur during pregnancy, childbirth, and the immediate postpartum period, timely receipt of medical services is considered to be life-saving [1,2]. Particularly, a package of evidence-based interventions known as emergency obstetric care (EmOC) is recommended by the World Health Organization (WHO) to treat obstetric complications, or acute conditions that can lead to maternal death, such as hemorrhage, eclampsia, sepsis, and obstructed labor [1,3]. In settings where the majority of women give birth at home in the absence of skilled birth attendants, receipt of EmOC is contingent upon timely care seeking by women and their families.

Care seeking (defined as activities undertaken to obtain treatment for health problems) is recognized as a complex behavioral process that is influenced by factors including demographic and socioeconomic characteristics, perceived need, and service availability [4-6]. In 2009, Gabrysch et al conducted a systematic review of literature published since the year 2000 on predictors of antenatal care use and delivery care in low-income settings [7]. Factors associated with facility use during delivery included maternal age greater than 18 years, antenatal care seeking, and nulliparity [7-11]. Higher socioeconomic status was correlated with increased utilization [12-15], while the association of female employment on maternal care utilization showed mixed results [11,15-18]. Women’s literacy has been consistently associated with maternity care use in the literature [9,19]. Some studies on care seeking have also analyzed perceived need factors, or factors that affect perception of whether care is needed for a health problem, including pregnancy wantedness and obstetric history [20,21]. Research from Peru and India suggests that women with wanted pregnancies were more likely to seek skilled attendance at delivery, with studies from rural Guatemala and sub-Saharan Africa showing associations between adverse obstetric history and skilled attendance at delivery [7,21,22].

In addition to demographic, socioeconomic, and perceived need factors that may affect care seeking for maternal health services in resource-poor settings, few studies have also considered aspects of service accessibility at health facilities, such as distance to facility and availability of services. A 2011 study from Zambia on determinants of care seeking during delivery showed that families were more likely (OR of 1.26; 95% CI of [1.07-1.48]) to visit facilities that offered comprehensive obstetric services compared to facilities that offered basic obstetric services only [23]. Chowdhury and colleagues demonstrated that if comprehensive obstetric services were not available at the nearest health facility, women in rural Bangladesh) bypassed their closest facilities for those that were located farther away with comprehensive obstetric services [24]. Yet these studies, focusing on service factors at health facilities, did not include other variables known to influence care seeking. Because obstetric complications represent acute life-threatening conditions, care seeking behaviors for these complications may differ from factors that influence care seeking for antenatal care or general health services. However, little data exist on care seeking behavior for obstetric complications by type of complication.

Care-seeking studies in Bangladesh have described a complex plural health system in which families typically visit multiple providers for care [25-29]. Data have shown that families in rural areas usually visit informal providers, such as village doctors, homeopathic providers, and traditional healers, before seeking formal providers such as doctors, nurses, midwives, or peripheral government workers for health care [4,25,30]. Although the WHO and most government health ministries recommend that women seek care from formal providers at health facilities for obstetric complications, research indicates that the majority of women in rural South Asian communities seek informal providers [4,30-34]. In plural health systems of South Asia, understanding the factors that influence care seeking from formal and informal providers remains important.

**Figure 1:**
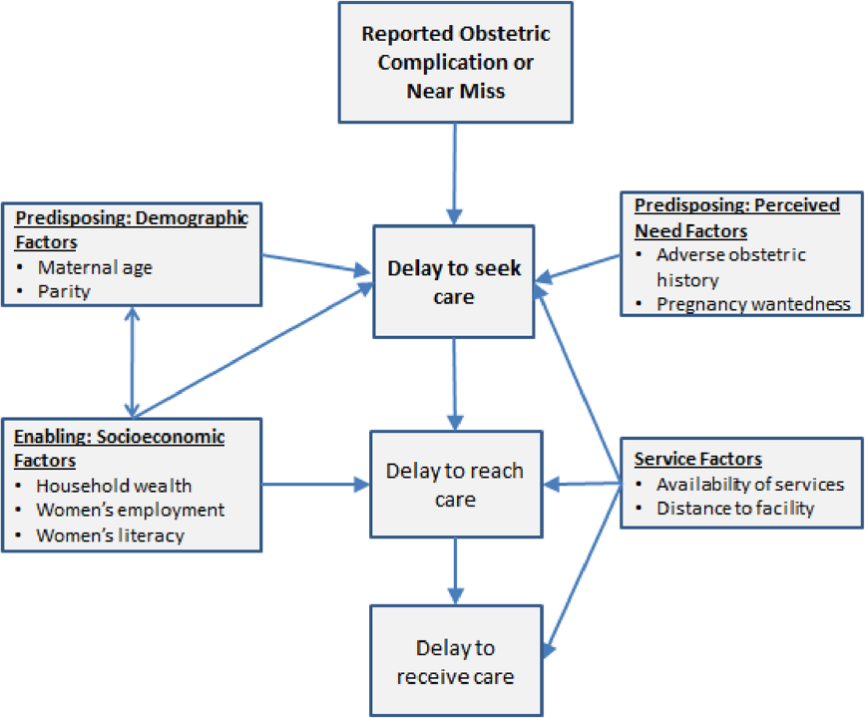
Ecological framework of factors associated with care seeking for emergency obstetric care. The framework shown in Figure 1 is adapted from the Three Delays Model [12] and Andersen’s Care-Seeking Framework [35]. Figure 1 shows the demographic, socioeconomic, perceived need, and service availability factors that are hypothesized to affect care seeking behavior for obstetric complications.

In this analysis, we explored socioeconomic, demographic, perceived need, and service factors associated with care seeking, by the type of complication reported. Our conceptual framework, based on the Three Delays Model and Andersen’s Care-Seeking Framework [12,35], suggests that predisposing (demographic factors and perceived need factors), enabling (socioeconomic factors), and service factors influence a person’s perceived benefits and barriers to undertake the action of care seeking (Figure 1). The objectives of this analysis are to describe care-seeking patterns for reported obstetric complications and near misses and to identify factors associated with care seeking, by type of complication.

## Methods

### Context of parent trial

Conducted in Gaibandha and Rangpur between 2007 and 2011, the JiVitA-3 community-randomized controlled trial enrolled 44,567 pregnant women to assess the effect of daily maternal antenatal supplementation with multiple micronutrients, compared to iron-folic acid, on six-month infant mortality (Clintrials.gov #NCT00860470) [36]. Mothers were instructed to take one tablet containing 15 micronutrients at a recommended daily allowance, from the first trimester through 12 weeks post-partum. The JiVitA-3 study area comprised 435 square kilometers of rural northwest Bangladesh, including 19 unions of Gaibandha and Rangpur Districts in Rangpur Division [37]. The study area was selected as representative of typical rural populations in Bangladesh based on population density (~1000 people per square kilometer), rural, agrarian characteristics, and economic and public health indicators [37]. As of 2010, Gaibandha District had a population of 2.3 million people, 51% of which was female and 49% male [38]. Literacy among females was reported at 63% [38].

Women were eligible for the parent trial if they could become pregnant (were of reproductive age 13-45 years, married and living with their husbands, not sterilized or menopausal, and whose husbands were not sterilized) [37]. Eligible women were visited by field workers every five weeks to administer pregnancy tests for missed menses. Women who were identified as pregnant based on urine tests were asked for consent to enroll in the parent trial.

Enrolled women were visited weekly by trained female workers to ascertain pregnancy outcomes (live births, stillbirths, induced abortions, or miscarriages). One month following their pregnancy outcomes, women were asked whether they experienced a series of morbidity symptoms in the 30 days before, during, or after their pregnancy outcome. The morbidity modules administered during these visits included structured morbidity symptoms comprising illnesses and conditions of pregnancy which were extensively pretested in the local language for comprehension. Women who reported experiencing any morbidity symptom for longer than one day were asked about the health provider from whom they sought care. Those who said that they felt that they nearly died at any time during pregnancy, delivery, or 30 days postpartum were considered near misses and asked about the symptoms they experienced and up to four providers from whom they sought care.

The JiVitA-3 study area was mapped using a Geographic Information System (GIS). More than 500,000 waypoints were collected on the location of participants’ households, formal health providers and facilities, landmarks, and roads. Changes or additions to households or landmarks were updated every five weeks [37].

The JiVitA-3 trial and all interim follow-up studies received ethical approval from the Bangladesh Medical Research Council (BMRC Reference Number 458) and the Johns Hopkins School of Public Health Institutional Review Board (IRB 00000570). The JiVitA-3 trial is registered under ClinicalTrials.gov NCT00860470.

### Classification of health care providers

Health providers visited for care were classified as “formal” or “informal.” A formal provider was defined as a health provider who was recognized and regulated by legally established authorities to provide health services [39]. In our study, formal providers included paramedics, family welfare assistants, family welfare visitors, medical assistants, health assistants, nurses, nurse-midwives, and MBBS (board-certified) doctors working at health facilities (family welfare centers, union health subcenters, NGO clinics, private clinics, sub-district health complexes, maternal and child welfare centers, and government hospitals). Informal providers were defined as health providers who were not legally authorized or regulated to provide health services in Bangladesh, including “village doctors” (*palli chikitsok*), traditional religious or spiritual healers such as *kabirajs, fakirs,* or *ojhas,* and homeopathic providers [40]. Our use of the term “formal” provider is synonymous with the terms “qualified” or “certified” provider as used in other analyses [4,25,41].

In this analysis, we focused on care-seeking behavior for conditions comprising obstetric complications or near misses. For women with obstetric complications, data on the last provider visited were collected, while data on all providers visited (up to four) were collected for near misses. We presented care-seeking patterns for women reporting obstetric complications and near misses separately due to the varying levels of information available on the number of health providers sought.

### Independent variables

Independent variables of interest were collected during enrollment interviews. Upon enrollment in the parent trial, women’s socioeconomic characteristics were assessed, including ownership of household assets such as the number of bicycles and construction of homes. To measure socioeconomic status, we used an asset-based wealth index constructed using principal component analysis of household assets and construction materials, as described elsewhere [42]. Enrolled women were also interviewed about their literacy, defined as the ability to read or write a letter in Bengali (the local language).

At enrollment, demographic data such as number of previous pregnancies and the outcome of these pregnancies were also collected. These data were used to define adverse obstetric history as report of an adverse outcome (stillbirth or abortion) during the previous pregnancy. Women’s age was self-reported at enrollment. Parity was defined as the number of births a woman reported. We included standard cutoffs used in the care-seeking literature to distinguish women at higher thresholds of risk for complications due to age (below 18 years, between 18 to 35 years, and greater than 35 years) and parity (nulliparous women).

During enrollment interviews, women were also asked whether they had wanted the index pregnancy, whether they had wanted the pregnancy later, or whether they had not wanted the pregnancy. Women who said they wanted their pregnancies later were classified as mistimed pregnancies, while women who said they had not wanted the pregnancy were classified as having unwanted pregnancies.

We collected primary data to assess the availability of obstetric services at the 14 health facilities most frequently visited for reported obstetric complications or near misses. This sample included all seven public facilities located in the two districts as well as the seven most frequently visited private clinics. The surveys consisted of interviews with health providers and administrators at health facilities on the availability of emergency obstetric services. Facilities were classified as providing comprehensive emergency obstetric services or basic emergency obstetric. Two main levels of care were defined: basic emergency obstetric care (BEmOC) and comprehensive emergency obstetric care (CEmOC), with BEmOC facilities providing basic obstetric services and CEmOC facilities providing basic obstetric services as well as C-sections and blood transfusions. Definitions were based on the 2010 WHO Service Availability and Readiness Assessment methodology [43]. Provision of C-sections was assessed on records of C-sections having been performed in the facility in past three months according to WHO Service Availability and Readiness Assessment definitions [43]. Additional information on facility surveys are provided elsewhere [44].

This analysis utilized extant data collected as part of the JiVitA-3 trial. To calculate distance to facility, the locations of health providers, households of study participants, and roads (based on data updated from June 30, 2011) were uploaded using ArcMap software [45]. R software [46] was used to assign values to roads based on difficulty of travel (paved or unpaved roads). Using this data, the most direct route-based paths from households to facilities based on the presence and type of road were calculated. These derived values of distance in kilometers were transferred to Stata software for inclusion in regression models. Distance to facility in kilometers was explored at cutpoints that appeared significant (<10 km, ≥10 km).

### Data analysis

We used multinomial logistic regression to understand factors influencing care seeking from both informal and formal providers. To construct regression models, we first checked data for completeness and accuracy and screened for missing values. Data analysis was performed using Stata 11 [47]. Multinomial logistic regression was used to obtain relative risk ratios and 95% confidence intervals to estimate the relationships of independent variables with the categorical outcome of care seeking. Women not seeking care were compared to women seeking informal or formal care, respectively. Women who reported visiting at least one formal provider were categorized in the formal care category, while women seeking informal providers only were classified in the informal group. Robust cluster estimates were generated to account for cluster-randomization in the parent trial. We retained independent variables strongly associated with care seeking in the literature (maternal literacy, age, and distance to facility) as well as potential confounders (year and season of pregnancy outcome) in the base model. Other variables were added in order of their effect sizes and significance in bivariate analysis, keeping and eliminating variables according to Akaike Information Criteria [48]. Potential interactions between covariates such as distance to facility and availability of services were explored and added to the model based on our theoretical framework.

As a sub-analysis, we used multinomial logistic regression to explore care from formal and informal providers by type of complication reported (eclampsia, sepsis, hemorrhage, or obstructed labor). We chose to adjust analyses by year and season of pregnancy outcome since we expected care-seeking behavior to change over time and to vary during seasons such as the monsoon season.

**Figure 2:**
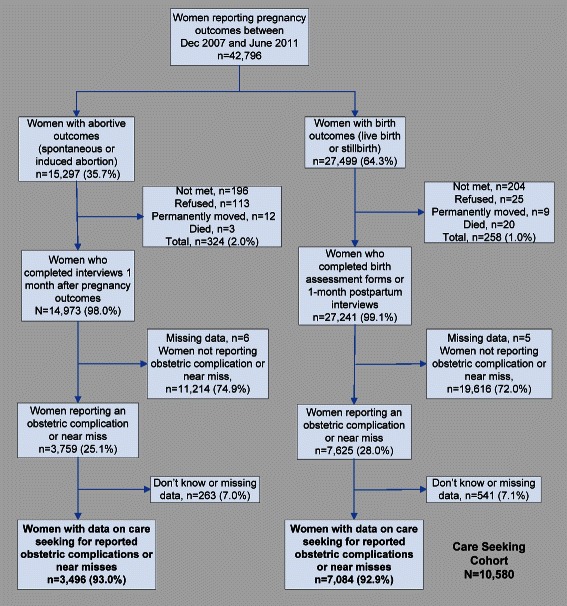
Analytic cohort (n = 10,580) between 2007 and 2011, by type of pregnancy outcome. Figure 2 shows the cohort for this analysis, comprised of married women of reproductive age with data on care seeking for reported obstetric complications or near misses between December 2007 and June 2011.

Between December 2007 and June 2011, 42,796 women reported pregnancy outcomes (Figure 2). Approximately 61% of women had live births, 3% had stillbirths, 25% had an induced abortion, and 11% had a spontaneous abortion. From the 11,384 women classified as reporting an obstetric complication or near miss, 93% (n = 10,580) had data on care seeking (including 9,576 women reporting obstetric complications and 1,004 women with near misses) and were included in the analytic cohort for this paper. Care-seeking patterns are presented separately for women with obstetric complications (n = 9,576) and women reporting near misses (n = 1,004) as different levels of information on providers sought were available for these two groups.

## Results

### Descriptive characteristics

Table 1 describes demographic, socioeconomic, and other characteristics of interest among the 10,580 women with data on care seeking. On average, 54% of women (n = 5,677) were literate and 44% (n = 5,150) were employed. The mean age at the time of pregnancy was 24.1 years (standard deviation: 6.6 years). Approximately 42% of women reported not wanting their pregnancy, while 14% reported mistimed pregnancies. Compared to women who did not seek care or who sought informal care, higher proportions of women who sought formal care were from the highest household wealth quartile (p < 0.001), literate (p < 0.001), employed (p < 0.001), and reported wanting their pregnancy (p < 0.001), among other factors.

**Table 1:** **Distribution of demographic, socioeconomic, and need characteristics among 10,580 women in rural Bangladesh**

Characteristics	No care (n = 2,319) %	Informal provider only (n = 5,639) %	At least one formal provider (n = 2,622) %	Total (n = 10,580) % (n)
*Demographic factors*				
Women’s age***				
<18 years	15.8	7.8	4.4	13.8 (1460)
18-35 years	77.5	78.1	77.2	77.0 (8147)
>35 years	6.7	14.0	18.4	9.1 (971)
Missing	0.0	0.1	0.0	0.1 (2)
Parity*				
Nulliparity	28.6	19.3	19.0	24.3 (2571)
1-2 births	46.4	48.4	48.0	47.9 (5068)
>2 births	25.0	32.3	32.9	27.8 (2941)
Missing	0.0	0.0	0.0	0.0 (0)
*Socioeconomic factors*				
Household wealth index ***				
Lowest Quartile	26.0	25.5	22.0	25.2 (2709)
2nd Quartile	25.3	25.6	22.9	25.0 (2661)
3rd Quartile	24.8	25.5	22.2	25.0 (2675)
Highest Quartile	23.6	23.2	32.8	24.6 (2519)
Missing	0.3	0.2	0.1	0.2 (16)
Women’s employment***				
No job	57.9	56.8	50.7	55.9 (5914)
Any job	42.0	43.1	49.2	44.0 (4653)
Missing	0.1	0.1	0.1	0.1 (13)
Women’s literacy***				
Illiterate	46.2	46.8	37.7	46.1 (4877)
Literate	53.5	53.0	62.2	53.7 (5686)
Missing	0.3	0.2	0.1	0.2 (17)
*Perceived need factors*				
**Obstetric history**				
Live birth or no previous Pregnancy	73.3	74.4	72.6	73.1 (7742)
Previous stillbirth or Abortion	26.7	25.5	27.4	26.8 (2835)
Missing	0.0	0.1	0.0	0.1 (3)
Pregnancy wantedness (women)***				
Wanted pregnancy	17.5	48.1	56.4	43.1 (4560)
Mistimed pregnancy	13.6	10.0	6.7	14.4 (1524)
Unwanted pregnancy	68.1	41.3	36.4	41.8 (4422)
Missing	0.8	0.6	0.5	0.7 (74)
*Service Factors*				
**Distance from facility (kilometers)**				
Less than 10 km	20.0	20.0	23.2	20.4 (2180)
≥10 km	78.9	79.2	75.8	78.6 (8315)
Missing	1.1	0.8	1.0	1.0 (85)

*p-value <0.05 of overall difference between the three groups using Chi-squared tests.

***p-value <0.001 of overall difference between the three groups using Chi-squared tests.

Table 1 shows characteristics of interest among 10,580 married women of reproductive age with data on care seeking for reported obstetric complications or near misses between December 2007 and June 2011.

### Care-seeking patterns

**Figure 3:**
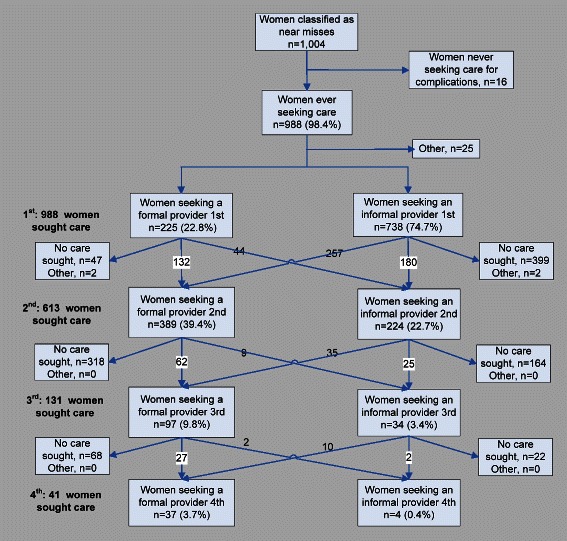
Care-seeking patterns among 1,004 women of reproductive age reporting near misses between 2007 to 2011. Data on up to four providers sought for care were available for women reporting near misses. Figure 3 shows the percentage and number of women seeking formal or informal care at initial stages and at later stages of care seeking. This figure illustrates the complex number and type of providers sought for care by women with life-threatening obstetric conditions.

Among the 9,576 women with data on care seeking for obstetric complications, approximately 77% (n = 7,338) sought any care, with 29% visiting at least one formal provider and 70% visiting informal providers only. Among women reporting near misses, 98% (n = 988) sought any care (Figure 3), with 23% (n = 225) seeking a formal provider initially and 75% (n = 738) seeking an informal provider initially. Figure 3 illustrates the complexity in care seeking for women with life-threatening conditions in the number and type of providers sought. Sixty-two percent of near misses (n = 631) sought multiple providers, with a large number of women (n = 257) visiting a formal provider after an initial visit to an informal provider. Seventy-five percent of near misses who sought care (n = 741) used a mobile phone to coordinate finances, transport, care, or to receive medical advice. Of the 482 near misses who sought formal care, 53% used non-motorized transport (bicycle rickshaw or flatbed van-rickshaw) to reach providers, whereas 47% (n = 330) used a motorized form of transport (auto-rickshaw, microbus, or ambulance) (data not shown).

Care-seeking patterns also differed significantly by type of complication. Fifty-seven percent of women reporting eclampsia (n = 177) sought care from at least one formal provider, while 28% of women reporting hemorrhage (n = 1,319), 22% reporting obstructed labor (n = 860), and 17% (n = 292) reporting sepsis sought care from at least one formal provider (p < 0.001). A higher proportion (30%) of women with live births sought health care from a formal provider compared to women who reported an induced abortion, spontaneous abortion, or stillbirth (19%; 21%, and 20%, respectively) (p < 0.001).

### Multivariable analyses

In the main adjusted model (Table 2), factors including maternal age less than 18 years and having a mistimed or unwanted pregnancy were negatively associated with care seeking. Women older than 35 years were significantly more likely to seek care (Table 2).

**Table 2:** **Multivariable multinomial logistic regression of factors associated with care seeking for reported obstetric complications** ^**a**^

	Sought informal provider		Sought formal provider	
Independent variables	Relative Risk Ratio (RRR)	95% Confidence Interval	Relative Risk Ratio (RRR)	95% Confidence Interval
**Demographic factors**				
*Women’s age 18-35 years (ref)*				
Age <18 years	0.81***	(0.68, 0.95)	0.78***	(0.64, 0.91)
Age >35 years	1.21***	(1.05, 1.37)	1.23***	(1.11, 1.35)
*Parity 1-2 (ref)*				
Nulliparity	0.90	(0.79, 1.02)	0.90	(0.76, 1.03)
Parity > 2	1.15	(0.97, 1.33)	1.15	(0.97, 1.33)
**Socioeconomic factors**				
*Lowest household wealth quartile (ref)*				
2^nd^ wealth quartile	1.01	(0.85, 1.17)	0.99	(0.86, 1.12)
3^rd^ wealth quartile	1.00	(0.82, 1.18)	0.99	(0.85, 1.13)
Highest wealth quartile	0.97	(0.82, 1.12)	1.49***	(1.33, 1.73)
*No employment (women) (ref)*				
Any employment (women)	0.97	(0.81, 1.12)	1.10*	(1.01, 1.18)
*Illiteracy (women) (ref)*				
Women’s literacy	1.00	(0.88, 1.13)	1.21**	(1.05, 1.42)
**Perceived need factors**				
*No adverse obstetric history (ref)*				
Adverse obstetric history	0.99	(0.85, 1.13)	1.04	(0.84, 1.18)
*Wanted pregnancy (women) (ref)*				
Mistimed pregnancy	0.86**	(0.74, 0.97)	0.83**	(0.71, 0.95)
Unwanted pregnancy	0.65***	(0.45, 0.86)	0.60***	(0.39, 0.83)
**Service factors**				
*Distance ≥ 10 km to facility (ref)*				
Distance <10 km to facility	1.00	(0.89, 1.10)	1.16**	(1.05, 1.28)
*Only BEmOC available at closest health facility (ref)*				
CEmOC available at closest health facility	1.02	(0.88, 1.17)	1.20**	(1.04, 1.36)

*p-value <0.05; **p-value <0.01; ***p-value <0.001.

^a^n = 5,516 for women seeking informal providers; n = 2,576 for women seeking formal providers; n = 2,488 for women not seeking care.

Table 2 shows adjusted relative risk ratios from multivariable multinomial logistic regression of socioeconomic, demographic, perceived need, and service factors with care seeking from formal and informal providers (compared to 2,226 women not seeking care) for reported obstetric complications and near misses among 10,318 married women of reproductive age in rural northwest Bangladesh from December 2007 to June 2011. Results are adjusted by year and season of pregnancy outcome and type of complication.

Socioeconomic variables including living in a household classified in the highest wealth quartile (RRR of 1.49; 95% CI of [1.33-1.73]), women’s literacy (RRR of 1.21; 95% CI of [1.05-1.42]), and women’s employment (RRR of 1.10; 95% CI of [1.01-1.18]), along with maternal age greater than 35 years and having a wanted pregnancy, were significantly and positively associated with care seeking from formal providers. None of the socioeconomic variables were significantly associated with care seeking from informal providers (Table 2).

Service factors also affected care seeking from formal providers. Living less than 10 kilometers from a health facility and having comprehensive services available at the nearest health facility were significantly associated with care seeking from formal providers (Table 2). The interaction of distance and availability (RRR of 1.07; 95% CI [1.03-1.13]) suggested that the association of distance on formal care seeking appeared to decrease with increased availability of comprehensive services at health facilities.

### Analyses by type of complication

For all complications, maternal age less than 18 years was negatively associated with care seeking, while maternal age greater than 35 years was positively associated with care seeking. For sepsis, obstructed labor, and hemorrhage, living in a household classified in the highest wealth quartile, women’s literacy, women’s employment, decreased distance to facility, and increased availability of services were positively associated with formal care seeking, while having a mistimed or unwanted pregnancy was negatively associated with care seeking (Table 3). For eclampsia, only maternal age and obstetric history were associated with care seeking from formal providers.

**Table 3:** **Multivariable multinomial logistic regression of factors associated with care seeking from formal and informal providers, by type of complication**

	Sought informal provider^a^				Sought formal provider^a^			
Independent variables	Eclampsia	Hemorrhage	Sepsis	Obstructed labor	Eclampsia	Hemorrhage	Sepsis	Obstructed labor
**Demographic factors**	**Relative Risk Ratio (RRR)**				**Relative Risk Ratio (RRR)**			
*Age 18-35 years (ref)*								
Age <18 years	0.70***	0.89***	0.87***	0.86***	0.73***	0.88***	0.90***	0.85***
Age >35 years	1.28***	1.15***	1.09***	1.12***	1.27***	1.15***	1.16***	1.11***
*Parity 1-2 (ref)*								
Nulliparity	0.90	0.91	0.92	0.89	0.88	0.90	0.91	0.86
Parity > 2 births	1.07	1.13	1.08	1.11	1.06	1.09	1.10	1.11
**Socioeconomic factors**								
*Lowest wealth quartile (ref)*								
2nd wealth quartile	0.98	0.99	0.96	0.95	0.97	0.98	0.96	0.96
3rd wealth quartile	0.99	0.97	0.97	0.99	0.97	0.96	0.98	0.98
Highest wealth quartile	1.05	1.06	1.05	1.07	1.03	1.38***	1.49***	1.26***
*No employment (women) (ref)*								
Any employment (women)	1.03	1.05	1.02	1.04	1.09	1.15**	1.11**	1.24**
*Illiteracy (women) (ref)*								
Women’s literacy	1.08	1.05	1.07	1.07	1.08	1.53***	1.77***	1.68***
**Perceived need factors**								
*No previous stillbirth or abortion (ref)*								
Adverse obstetric history	1.10	1.08	1.03	1.11	1.19**	1.04	1.11	1.14
*Wanted pregnancy (women) (ref)*								
Mistimed pregnancy	0.91	0.90**	0.87**	0.82**	0.93	0.88**	0.85**	0.87**
Unwanted pregnancy	0.94	0.67***	0.60***	0.71***	0.92	0.64***	0.73***	0.61***
**Service factors**								
*Distance ≥ 10 km to facility (ref)*								
Distance <10 km to facility	1.00	1.01	0.98	1.02	1.04	1.16**	1.19**	1.18**
*Only BEmOC at facility (ref)*								
CEmOC available at facility	1.02	1.00	1.02	0.99	1.03	1.11**	1.31**	1.19**

**p-value <0.01; ***p-value <0.001.

^a^n = 294 for eclampsia; n = 3,002 for hemorrhage; n = 2,386 for sepsis; n = 2,410 for obstructed labor; n = 2,488 for women not seeking care.

Table 3 shows adjusted odds ratios from multivariable multinomial logistic regression of socioeconomic, demographic, perceived need, and service factors with care seeking from formal providers and informal providers, by type of complication reported, among married women of reproductive age in rural northwest Bangladesh from December 2007 to June 2011. Results are adjusted by year and season of pregnancy outcome.

## Discussion

Although the majority of women sought care for reported complications, only a quarter of women visited formal providers and the majority of women sought care from informal providers. Factors such as maternal age less than 18 years and unwanted or mistimed pregnancy discouraged care seeking, while socioeconomic factors were associated with care seeking from formal providers. Short distance to facility and increased availability of services, along with improved socioeconomic status, women’s literacy, and women’s employment appeared to influence care seeking from formal providers. Higher percentages of women sought formal care for eclampsia compared to complications of sepsis, hemorrhage, and obstructed labor.

Our analyses, showing that 77% of women reporting complications sought any care, are similar to the care-seeking patterns presented in other studies of delivery-related care in rural Bangladesh. Using data from the 2001 Bangladesh Maternal Mortality and Health Care Survey, Koenig et al showed that approximately 71% (n = 3,939) of women who reported complications during pregnancy sought care [4]. The 2007 BDHS reported that, among a sample of 711 women reporting complications during a delivery in the past three years, 80% sought care [31]. Few previous studies have explored care-seeking patterns by type of complication and reported severity. In our study, a higher proportion of women (48%) sought formal care for near misses that they considered to be life-threatening than for obstetric complications (29%).

Care-seeking patterns also differed by type of complication, with more than half of women with reported eclampsia (57%) seeking formal care compared to less than a third of women reporting sepsis, obstructed labor, or hemorrhage. These differences in proportions of women seeking formal care may be due to severity or perceived severity of conditions. Compared to other obstetric complications, eclampsia has a higher case-fatality ratio and is more rare [49]. Community-based research on obstetric complications has suggested that convulsions, the main symptom of eclampsia, may be distinctive and recognized by families as requiring medical attention [41,50,51]. However, symptoms comprising sepsis and obstructed labor, high fever and length of labor pains, are more generalized and may not be readily recognized by families as necessitating medical care [4,41,50-53]. Community-based programs that aim to increase household awareness of obstetric conditions requiring health care may consider refining messages on symptoms of sepsis, hemorrhage, or obstructed labor to increase recognition of severity. In South Asia contexts, studies suggest that beliefs surrounding pregnancy and childbirth, such as the idea that pregnant women are “polluted” with impurities, may discourage recognition of postpartum bleeding [28,41,49,54]. For example, Sibley et al have shown that untrained birth attendants in rural Bangladesh and India considered excessive bleeding after childbirth to be normal in order to purge the body of “pollution” [54,55]. Programs may also consider targeting awareness campaigns to dispel misconceptions surrounding pregnancy and childbirth which may discourage report of complications [49-51].

Maternal age was associated with care seeking from both formal and informal providers for reported obstetric complications and near misses. Compared to adolescents, women older than 18 years have been shown to have more influence in household decisions regarding health care [11,15,52]. Adolescents tend to have lower status in families, and may have lower value placed on their health as well as higher risk for complications and maternal death compared to older women [11,15,52,56]. A 2014 review of data from 144 countries suggests a slightly elevated risk of maternal mortality among adolescents aged 15-19 compared to women aged 20-24 years [57]. Adolescents may utilize services less than women in other age groups. Even among adolescents who admitted to health facilities for delivery, a 2014 multi-country survey found that adolescents had lower coverage of prophylactic uterotonics, prophylactic antibiotics, and antenatal corticosteroids, and had infants with worse outcomes (prematurity, low birth weight, and severe neonatal conditions compared to women in other age groups [58]. These factors may further discourage younger women from seeking health care for health problems.

Pregnancy wantedness was also significantly associated with care seeking from formal and informal providers. When pregnancies are wanted, data suggest that families are more likely to engage in optimal care-seeking behaviors during pregnancy [20-22,55,59]. In our study, women who reported that their pregnancies were mistimed or unwanted were less likely to seek care for obstetric complications. The 2011 Bangladesh DHS shows that the top reason listed by women for contraceptive discontinuation and switching was concern about side effects [31]. Counseling on family planning may help to promote contraceptive continuity by addressing concerns about side effects and promoting discussion of contraceptive use among couples. In our analysis, a lower proportion of women who reported complications associated with induced abortions sought care from formal providers compared to women with live births, possibly due to the stigma surround induced abortions in this setting [60,61]. Although menstrual regulation is legal in Bangladesh, studies suggest that provider bias and community norms may discourage care seeking for related complications [60-63]. This analysis suggests the continued need for programs for prevention of unwanted pregnancies and promotion of care seeking for all women with obstetric complications.

Identifying factors that affect formal care seeking is important since the WHO recommends that women experiencing obstetric complications seek medical care from providers who are authorized and regulated to provide health services [63]. In this analysis, improved socioeconomic status appeared to enable care seeking from formal providers for reported obstetric complications or near misses. Research in Bangladesh have shown a significant wealth disparity in maternal care utilization [34,64], with numerous studies indicating increased care seeking for maternal services with improved wealth status [65,66]. Female literacy and employment have been considered as proxies of women’s empowerment and autonomy for reproductive behavior and health care [8,10]. Research has shown that women’s contribution to household income has been tied to greater decision-making power regarding health care [67,68].

Informal providers continue to play an important role in first-line care for obstetric complications experienced by this study population. Half of all women reporting complications visited informal providers in this analysis, while less than a third sought care from formal providers. Among women reporting life-threatening near misses, 75% of women first sought informal providers. Moreover, socioeconomic factors were not significantly associated with seeking informal providers. As trusted members of their community who are often visited for initial care, qualitative studies have suggested that informal providers may be well-positioned to facilitate referral of women with complications to formal providers [64,69,70]. Future studies to date could assess the ability of different cadres of informal providers to provide diagnosis and referral for obstetric complications in community settings.

Factors affecting service accessibility and provision at health facilities were also important in the decision to seek formal rather than informal care. Decreased distance to facility appeared to significantly improve care seeking from formal providers among women reporting sepsis, hemorrhage, and obstructed labor. Distance to facility is considered to capture poor road infrastructure, poor communication between communities, and other aspects of remoteness that are difficult to measure quantitatively [7]. The availability of comprehensive emergency obstetric care services at health facilities, including C-section and blood transfusion, improved care seeking from formal providers. Families appeared to be willing to travel greater distances for health facilities that offered comprehensive services. Improving the level of services at facilities may help to decrease the delay to seek formal care during obstetric crises.

### Limitations

Because the data in the parent trial were not exclusively collected to determine independent variables of care-seeking behavior, we were unable to make direct causal inferences. However, the presented associations exhibited temporal relationships and coherence with previous studies. In our analyses, we were unable to include some independent variables known to be important for care seeking (degree of social network and social support, awareness of delivery complications, and perceived quality of care) since these data were not collected in the parent trial. While this study provided information on distance between women’s homes and health facilities, data on distance from homes to informal providers were not collected. In addition, data on the specific relationship of relatives who served as informal providers to women who sought care were not collected.

## Conclusions

This analysis used prospective data on structured symptoms comprising obstetric complications and near misses, assessed within two to eight weeks to minimize recall bias. Detailed morbidity modules facilitated the exploration of results by complication (hemorrhage, obstructed labor, sepsis, and eclampsia). Data on socioeconomic, demographic, need, and service availability factors were collected and available for analysis. The large sample size provided sufficient power for main analyses and sub-analysis. Information on updated locations of participants and health providers, available from an extensive Geographic Information System, allowed for calculation of distance to facility.

Our analyses of care-seeking behavior by type of complication and severity represent unique contributions to the literature on care seeking for obstetric complications. Further study on factors affecting care seeking, by type of complication, may inform programs that aim to improve care-seeking behavior among households for obstetric complications. Future research could focus on optimal content of messages and optimal delivery of messages to improve recognition of symptoms requiring medical attention.

Care seeking in rural, population-dense communities reflects a complex behavioral process in response to demographic and socioeconomic factors, perceived need, and service availability. Bangladesh’s current maternal health strategy focuses on improving service availability at health facilities in order to increase utilization of obstetric care. While improved service availability was shown to increase formal care seeking, our findings suggest the need for a comprehensive strategy that also addresses socioeconomic, demographic, and perceived need factors that may affect formal care seeking. Along with improved service availability, policies to improve literacy among women, reduce early marriage, and reduce unmet need for family planning may help to promote care seeking from formal providers. While continuing to support safe motherhood strategies that promote provision of emergency obstetric services by formal providers, programs may utilize the ubiquity of first-line informal providers to facilitate referral for obstetric complications. In conjunction with improved geographic distribution of health facilities and increased service availability at facilities, policymakers should also consider programs that address socioeconomic disparities and optimize community-based messaging on symptoms to promote care seeking for obstetric complications in rural communities of Bangladesh.
